# Supplementary material for: Combined Peptidomics and Metabolomics Analyses to Characterize the Digestion Properties and Activity of Stropharia rugosoannulata Protein–Peptide-Based Materials
Source: Foods. 2024 Aug 15;13(16):2546. doi: 10.3390/foods13162546 (PMC11353256; doi:10.3390/foods13162546)
Supplement: Supplementary file 1 [file foods-13-02546-s001.zip › foods-3125848-supplementary/foods-3125848-supplementary figures.pdf]

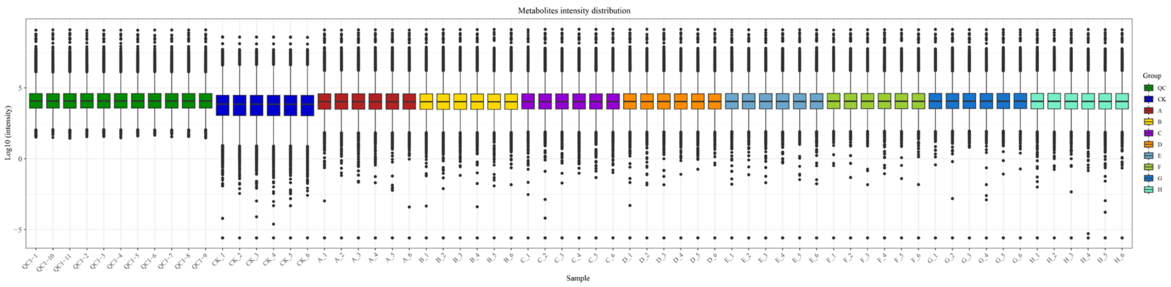

Figure S1. Box line diagram for stability assessment of sample metabolomics mass spectrometry assay

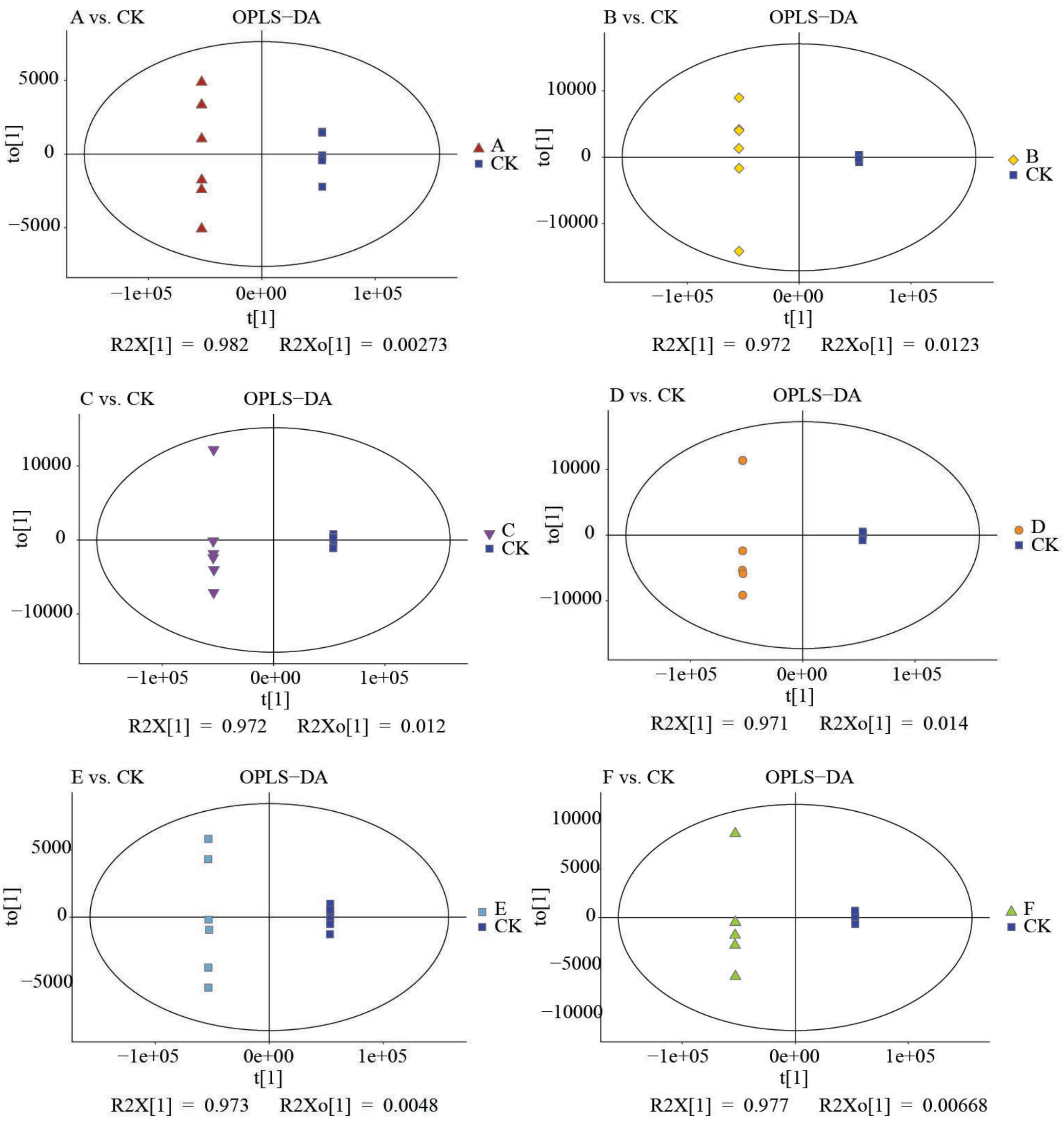

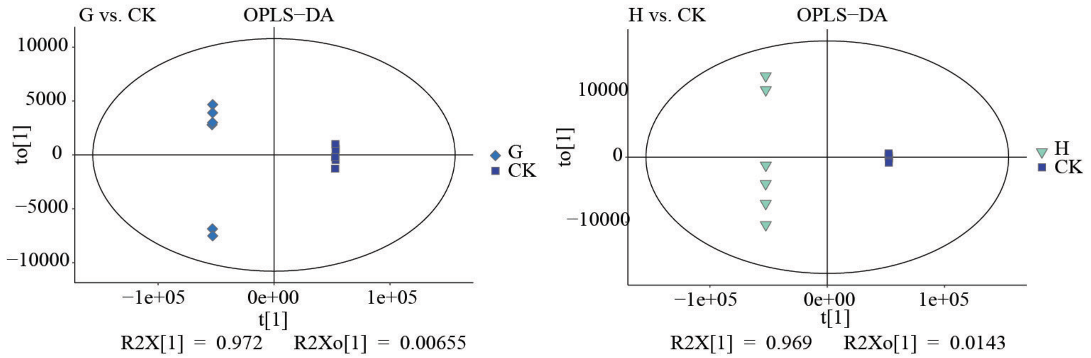

Figure S2. OPLS-DA analysis of digestion products

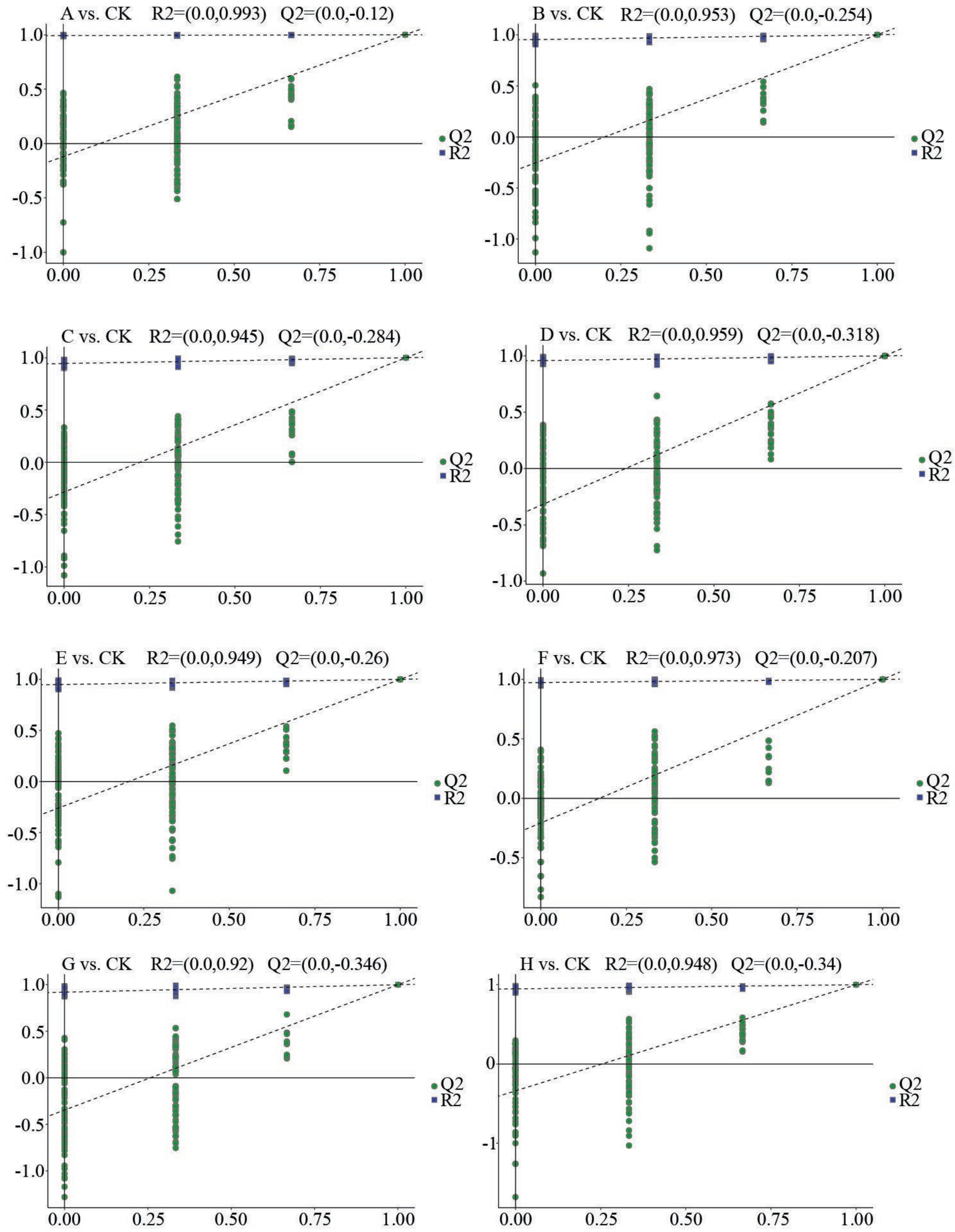

Figure S3. OPLS-DA model validity response permutation testing plots

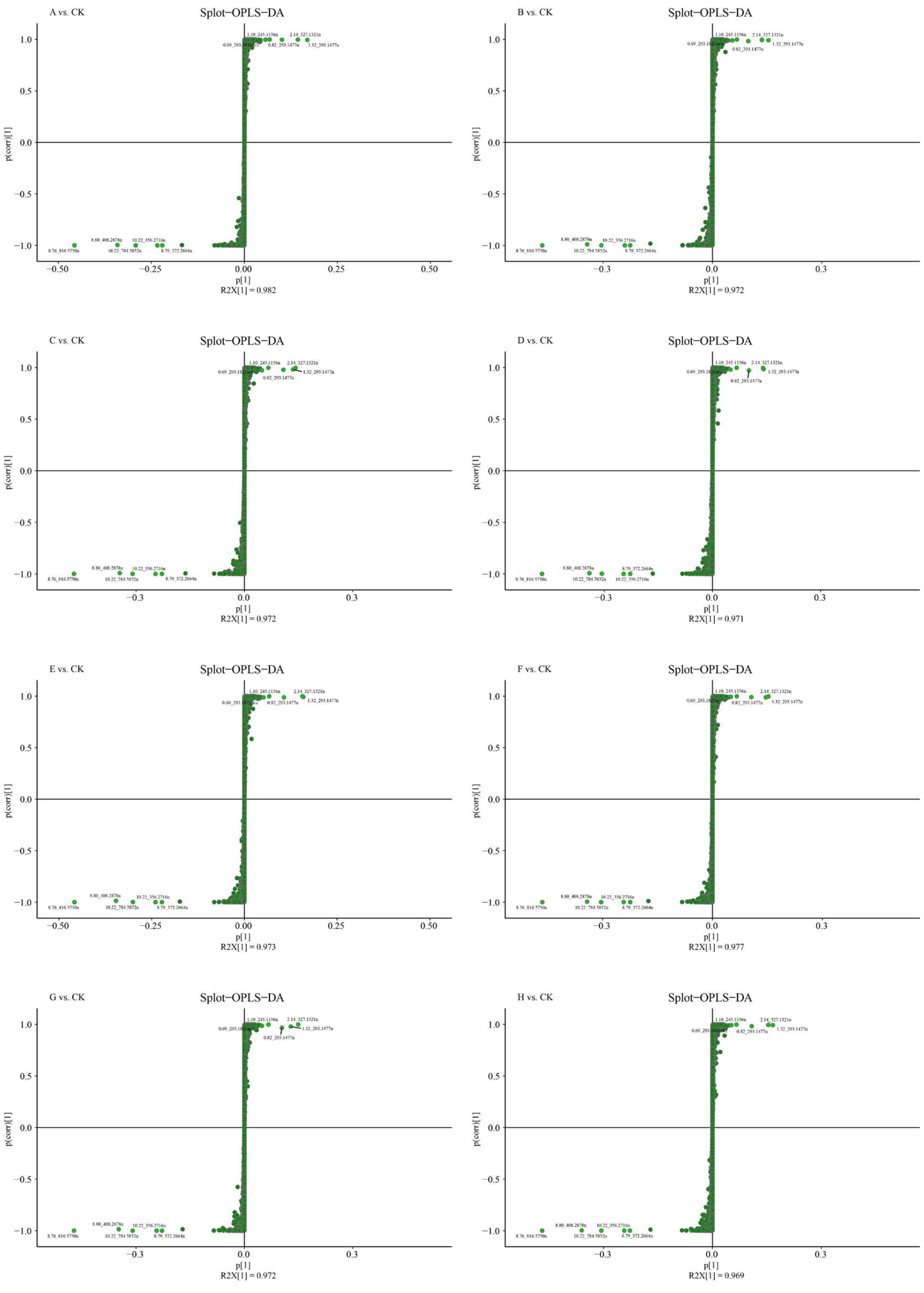

Figure S4. Metabolite distribution S-plots for sample comparison groups

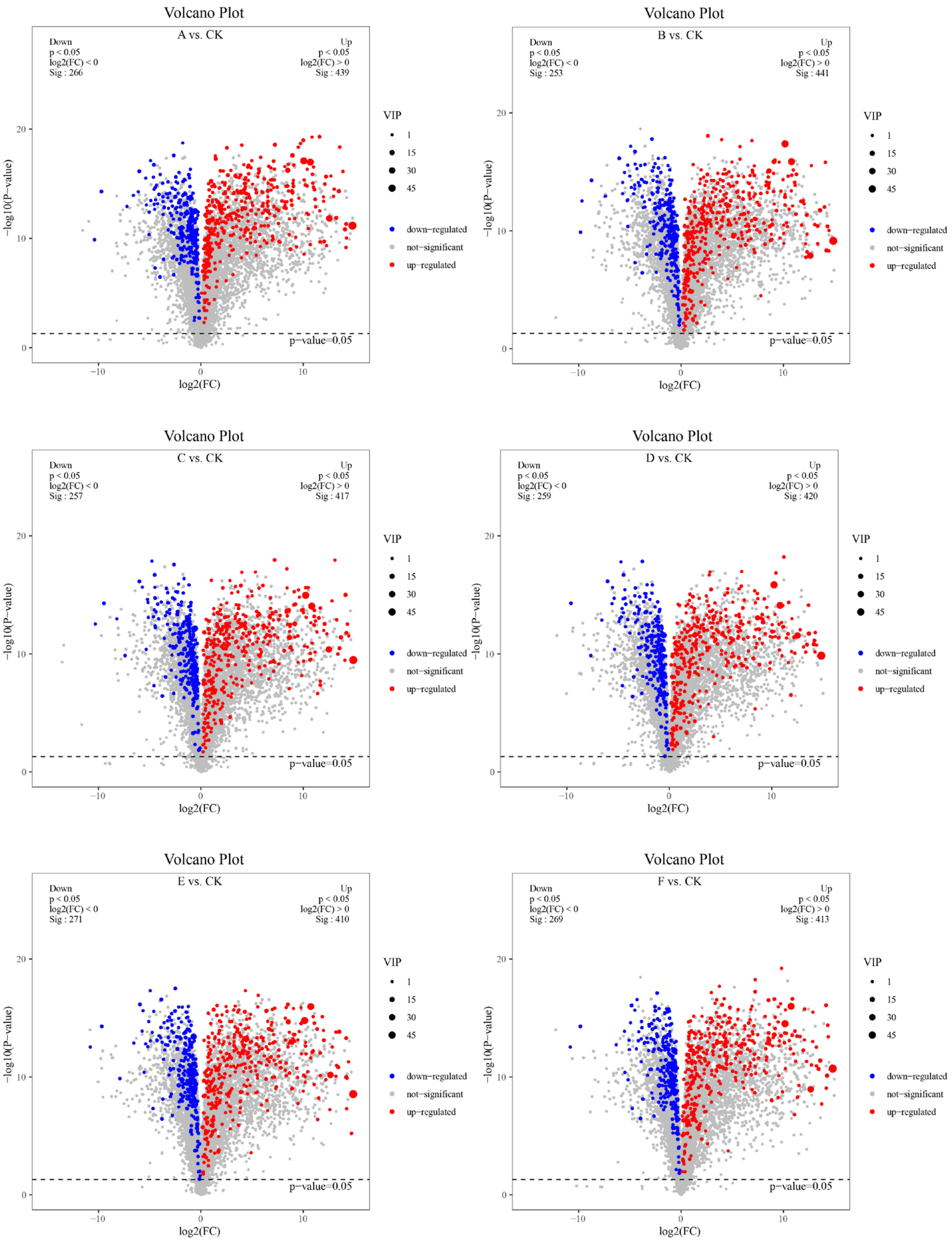
Volcano Plot
A vs. CK
Down
p < 0.05
log2(FC) < 0
Sig : 266
Up
p < 0.05
log2(FC) > 0
Sig : 439
p−value=0.05
−log10(P−value)
log2(FC)
VIP
1
15
30
45
down−regulated
not−significant
up−regulated
Volcano Plot
B vs. CK
Down
p < 0.05
log2(FC) < 0
Sig : 253
Up
p < 0.05
log2(FC) > 0
Sig : 441
p−value=0.05
−log10(P−value)
log2(FC)
VIP
1
15
30
45
down−regulated
not−significant
up−regulated
Volcano Plot
C vs. CK
Down
p < 0.05
log2(FC) < 0
Sig : 257
Up
p < 0.05
log2(FC) > 0
Sig : 417
p−value=0.05
−log10(P−value)
log2(FC)
VIP
1
15
30
45
down−regulated
not−significant
up−regulated
Volcano Plot
D vs. CK
Down
p < 0.05
log2(FC) < 0
Sig : 259
Up
p < 0.05
log2(FC) > 0
Sig : 420
p−value=0.05
−log10(P−value)
log2(FC)
VIP
1
15
30
45
down−regulated
not−significant
up−regulated
Volcano Plot
E vs. CK
Down
p < 0.05
log2(FC) < 0
Sig : 271
Up
p < 0.05
log2(FC) > 0
Sig : 410
p−value=0.05
−log10(P−value)
log2(FC)
VIP
1
15
30
45
down−regulated
not−significant
up−regulated
Volcano Plot
F vs. CK
Down
p < 0.05
log2(FC) < 0
Sig : 269
Up
p < 0.05
log2(FC) > 0
Sig : 413
p−value=0.05
−log10(P−value)
log2(FC)
VIP
1
15
30
45
down−regulated
not−significant
up−regulated

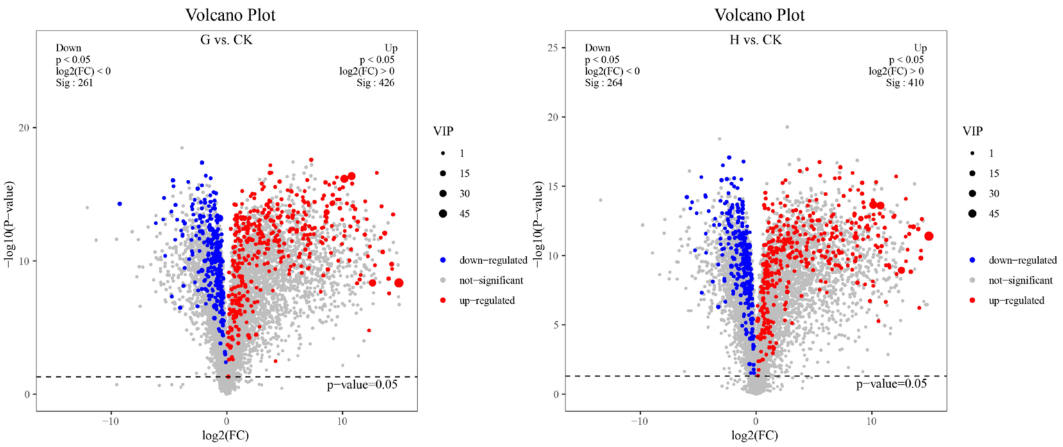

Figure S5. Metabolite distribution volcano plots for sample comparison groups

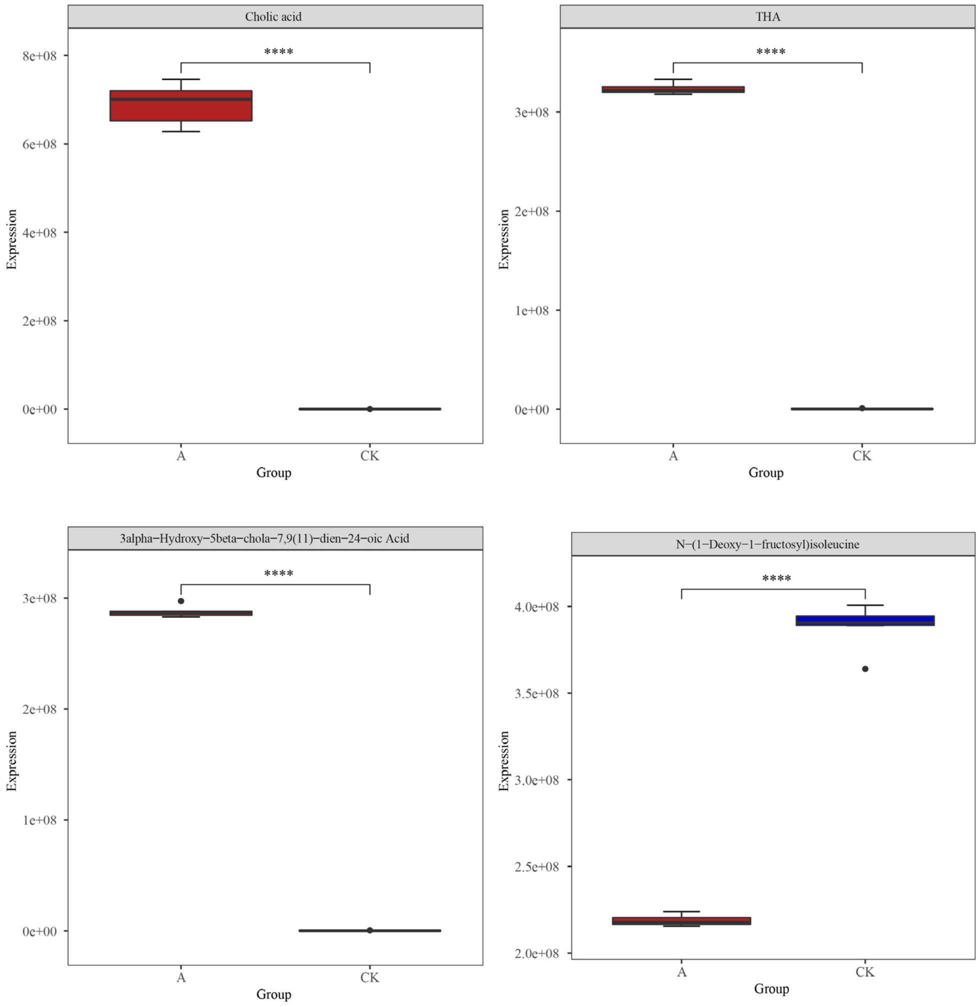

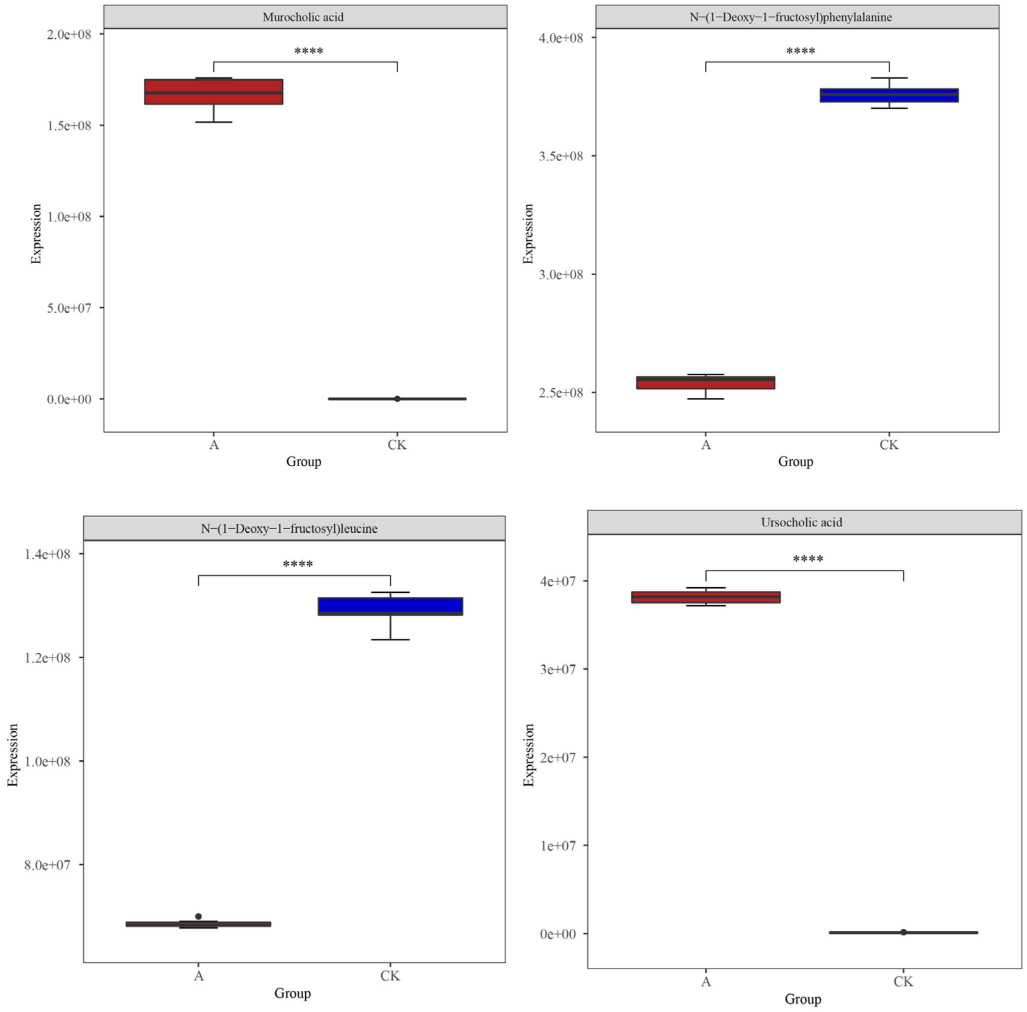

Murocholic acid
Expression
2.0e+08
1.5e+08
1.0e+08
5.0e+07
0.0e+00
****
A
CK
Group
N−(1−Deoxy−1−fructosyl)phenylalanine
Expression
4.0e+08
3.5e+08
3.0e+08
2.5e+08
****
A
CK
Group
N−(1−Deoxy−1−fructosyl)leucine
Expression
1.4e+08
1.2e+08
1.0e+08
8.0e+07
****
A
CK
Group
Ursocholic acid
Expression
4e+07
3e+07
2e+07
1e+07
0e+00
****
A
CK
Group

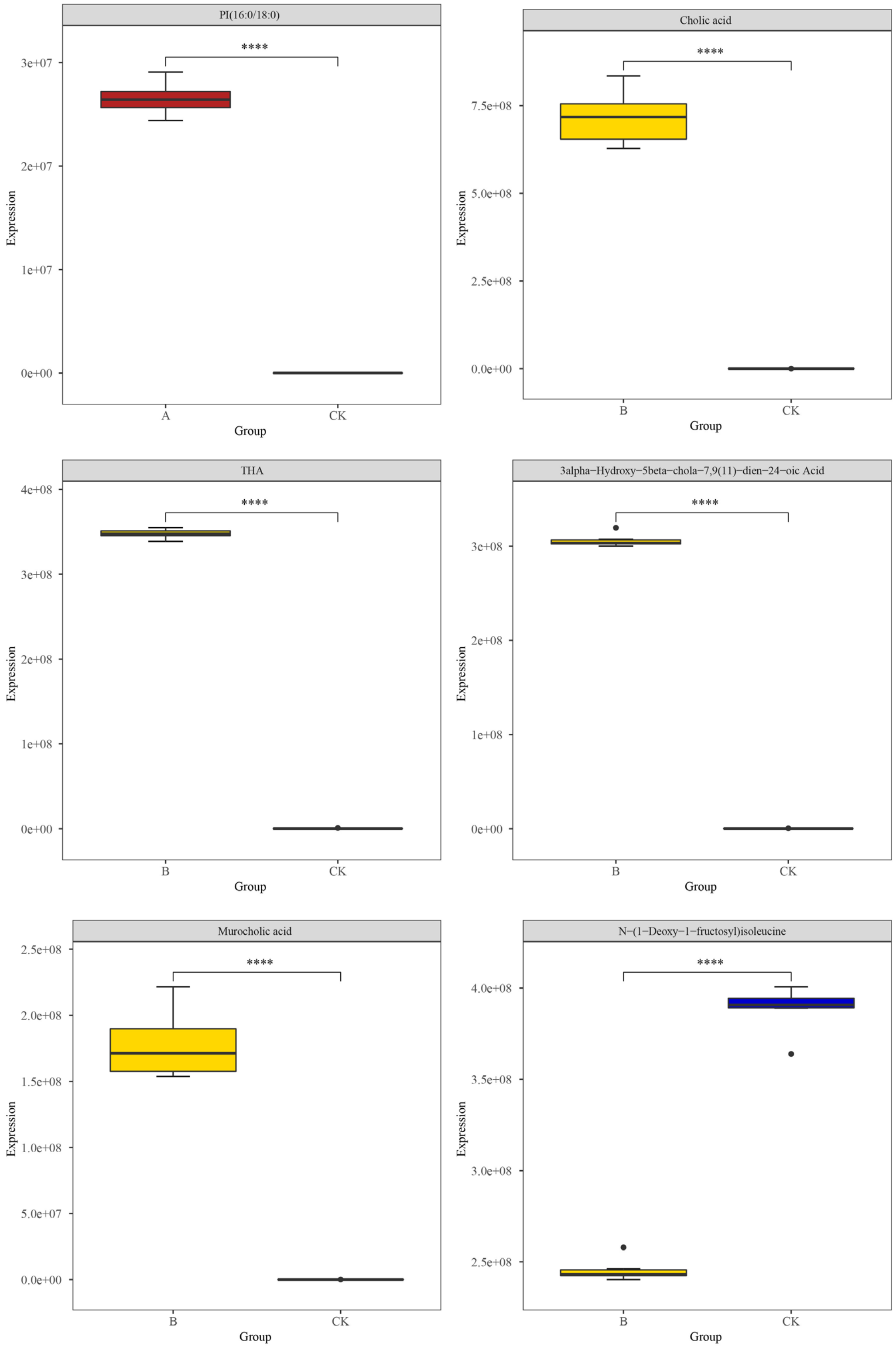
PI(16:0/18:0)
****
Expression
3e+07
2e+07
1e+07
0e+00
A
CK
Group
Cholic acid
****
7.5e+08
5.0e+08
2.5e+08
0.0e+00
B
THA
4e+08
3e+08
2e+08
1e+08
3alpha−Hydroxy−5beta−chola−7,9(11)−dien−24−oic Acid
Murocholic acid
2.5e+08
2.0e+08
1.5e+08
1.0e+08
5.0e+07
N−(1−Deoxy−1−fructosyl)isoleucine
4.0e+08
3.5e+08
3.0e+08

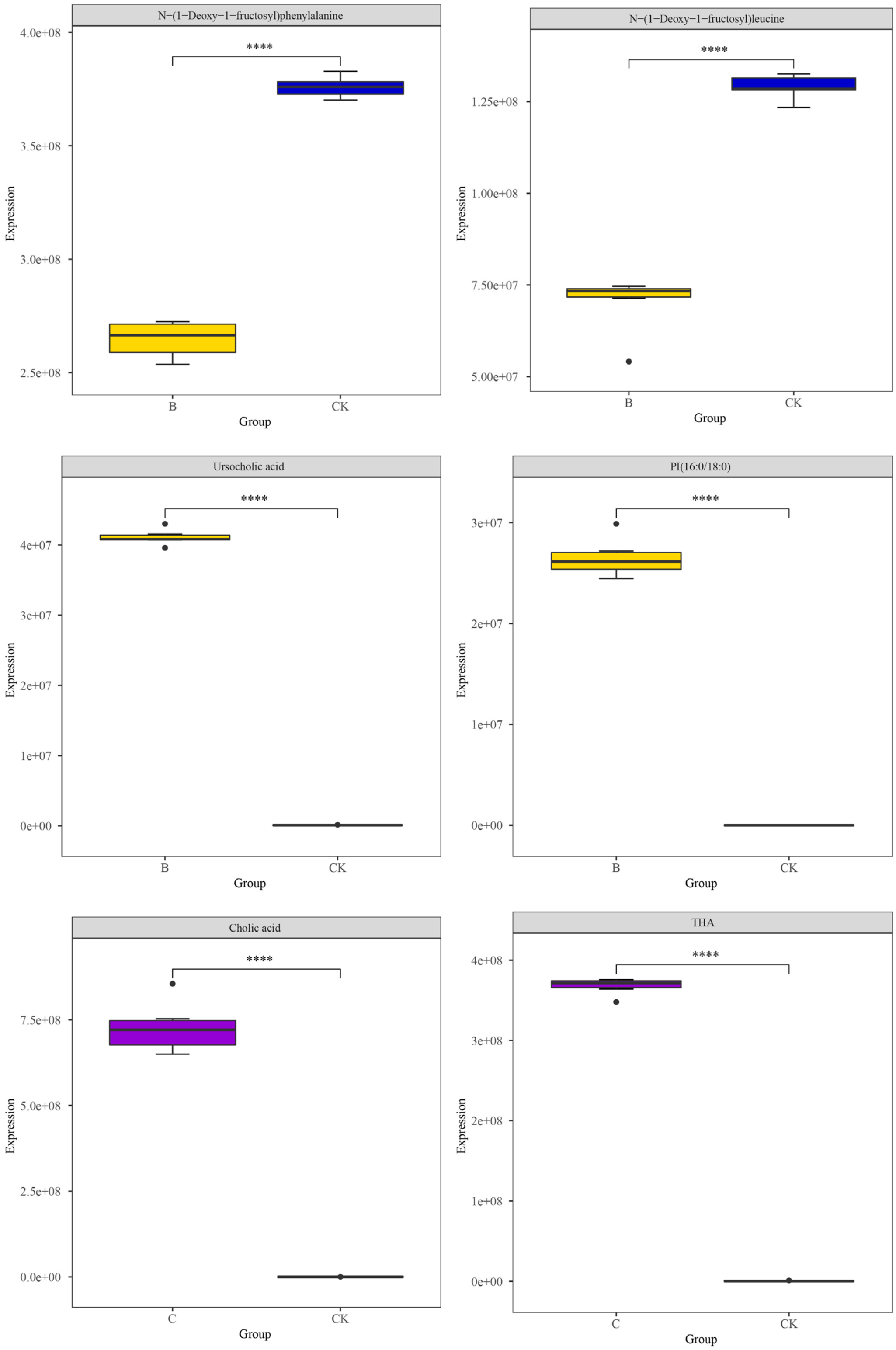
N−(1−Deoxy−1−fructosyl)phenylalanine
****
4.0e+08
3.5e+08
3.0e+08
2.5e+08
Expression
B
CK
Group
N−(1−Deoxy−1−fructosyl)leucine
****
1.25e+08
1.00e+08
7.50e+07
5.00e+07
Expression
B
CK
Group
Ursocholic acid
****
4e+07
3e+07
2e+07
1e+07
0e+00
Expression
B
CK
Group
PI(16:0/18:0)
****
3e+07
2e+07
1e+07
0e+00
Expression
B
CK
Group
Cholic acid
****
7.5e+08
5.0e+08
2.5e+08
0.0e+00
Expression
C
CK
Group
THA
****
4e+08
3e+08
2e+08
1e+08
0e+00
Expression
C
CK
Group

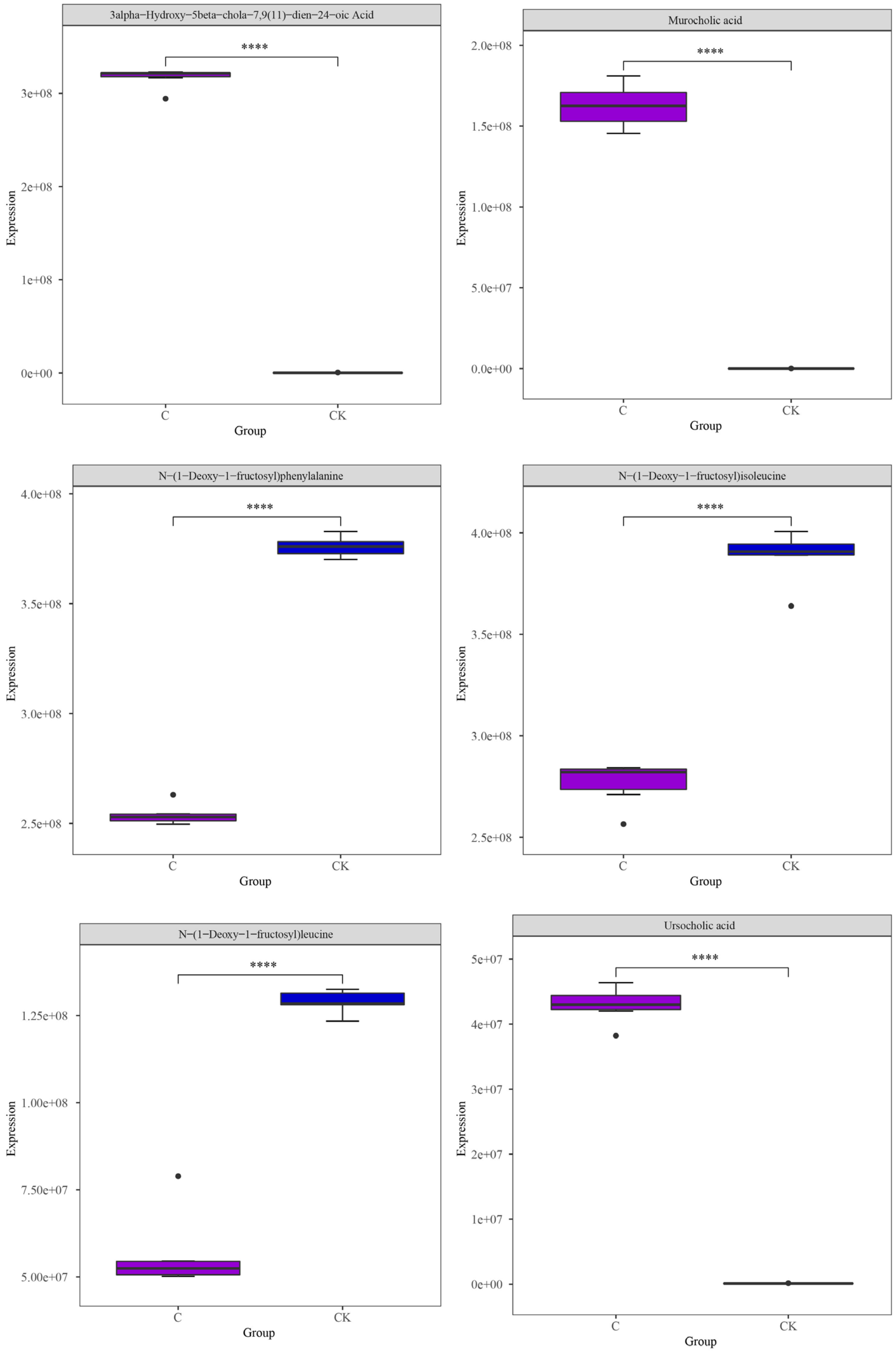
3alpha−Hydroxy−5beta−chola−7,9(11)−dien−24−oic Acid
****
3e+08
2e+08
1e+08
0e+00
Expression
C
CK
Group
Murocholic acid
****
2.0e+08
1.5e+08
1.0e+08
5.0e+07
0.0e+00
Expression
C
CK
Group
N−(1−Deoxy−1−fructosyl)phenylalanine
****
4.0e+08
3.5e+08
3.0e+08
2.5e+08
Expression
C
CK
Group
N−(1−Deoxy−1−fructosyl)isoleucine
****
4.0e+08
3.5e+08
3.0e+08
2.5e+08
Expression
C
CK
Group
N−(1−Deoxy−1−fructosyl)leucine
****
1.25e+08
1.00e+08
7.50e+07
5.00e+07
Expression
C
CK
Group
Ursocholic acid
****
5e+07
4e+07
3e+07
2e+07
1e+07
0e+00
Expression
C
CK
Group

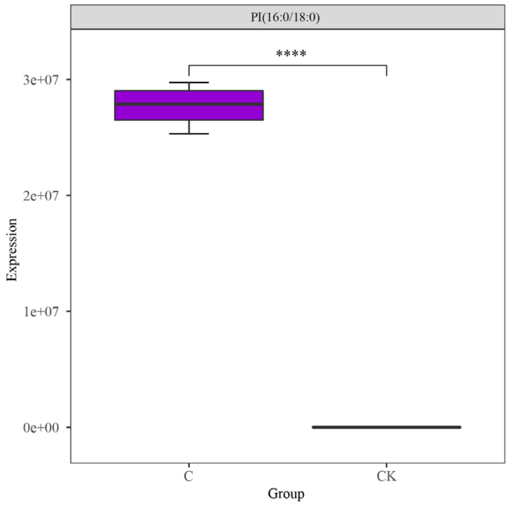
PI(16:0/18:0)
****
3e+07
2e+07
1e+07
0e+00
Expression
C
CK
Group

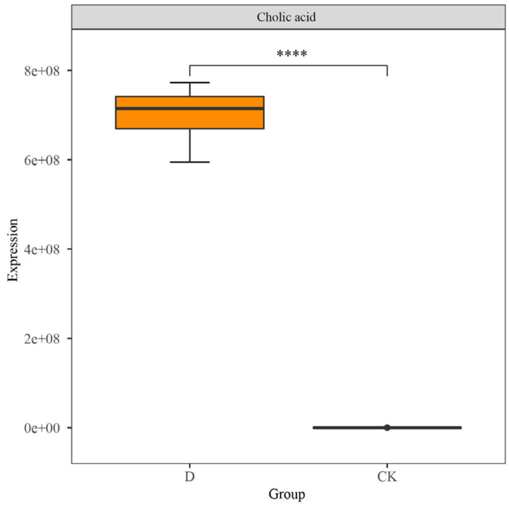
Cholic acid
****
8e+08
6e+08
4e+08
2e+08
0e+00
Expression
D
CK
Group

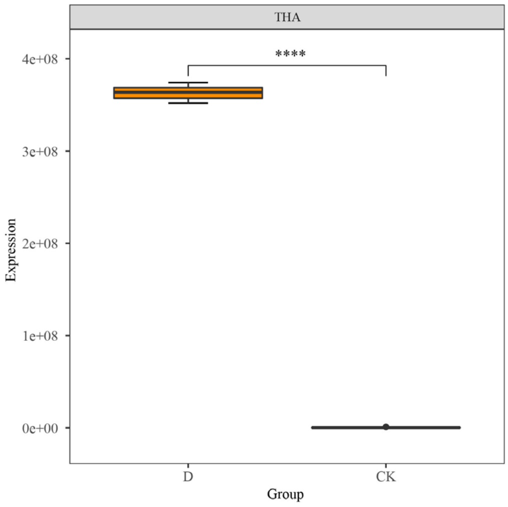
THA
****
4e+08
3e+08
2e+08
1e+08
0e+00
Expression
D
CK
Group

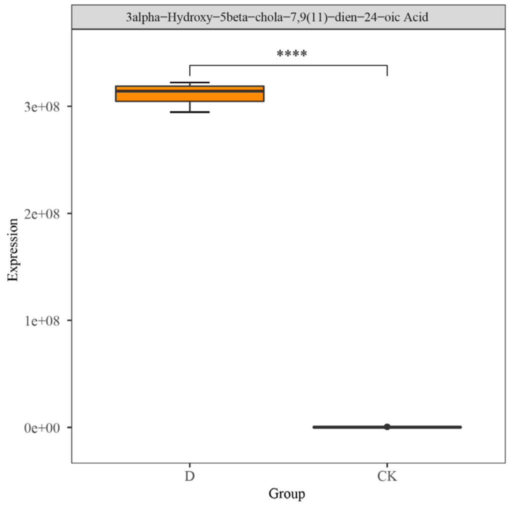
3alpha−Hydroxy−5beta−chola−7,9(11)−dien−24−oic Acid
****
3e+08
2e+08
1e+08
0e+00
Expression
D
CK
Group

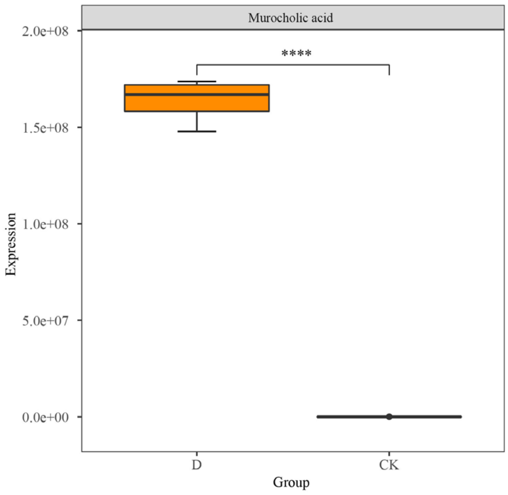
Murocholic acid
****
2.0e+08
1.5e+08
1.0e+08
5.0e+07
0.0e+00
Expression
D
CK
Group

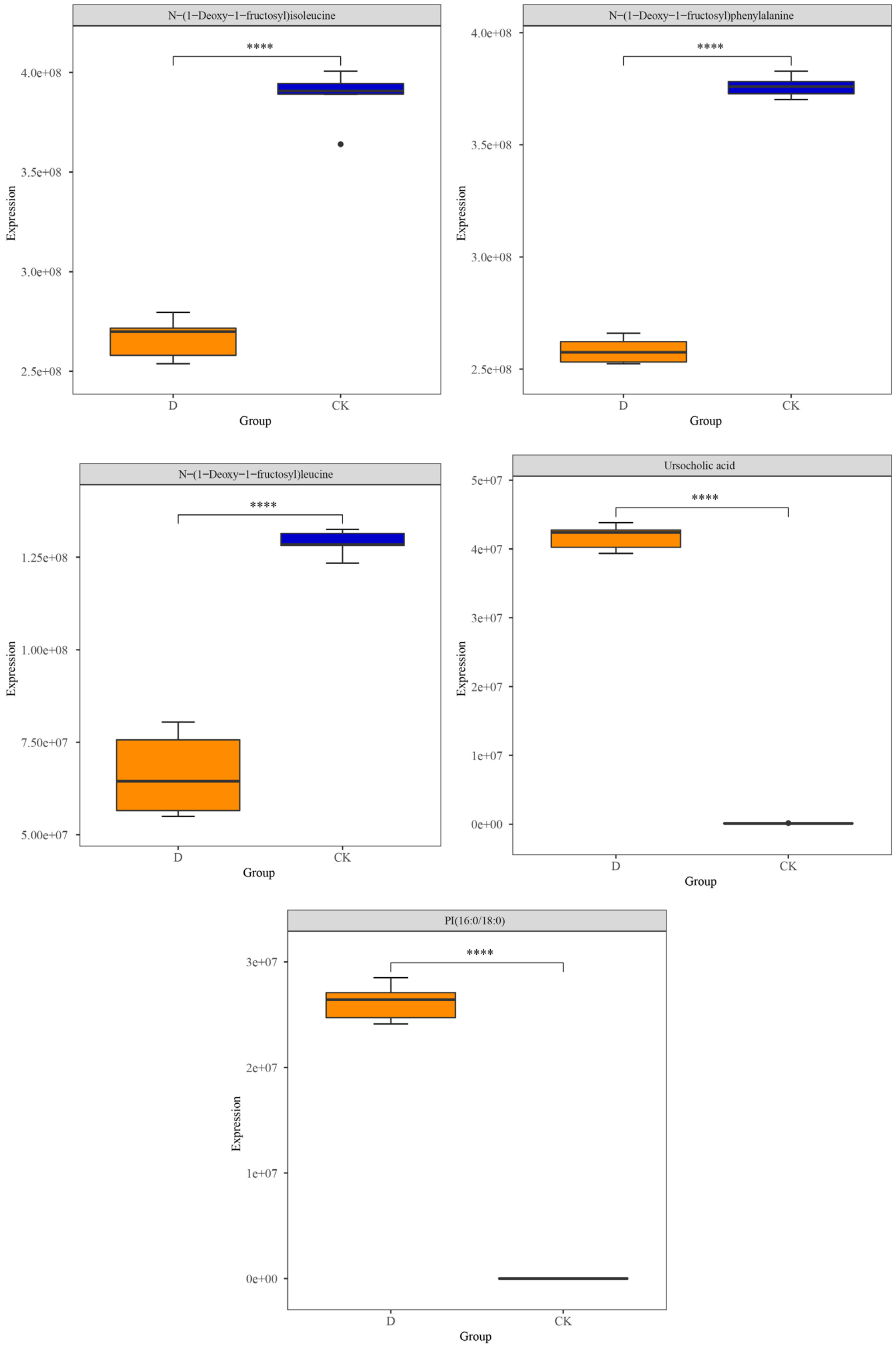

N−(1−Deoxy−1−fructosyl)isoleucine
****
4.0e+08
3.5e+08
3.0e+08
2.5e+08
Expression
D
CK
Group
N−(1−Deoxy−1−fructosyl)phenylalanine
****
4.0e+08
3.5e+08
3.0e+08
2.5e+08
Expression
D
CK
Group
N−(1−Deoxy−1−fructosyl)leucine
****
1.25e+08
1.00e+08
7.50e+07
5.00e+07
Expression
D
CK
Group
Ursocholic acid
****
5e+07
4e+07
3e+07
2e+07
1e+07
0e+00
Expression
D
CK
Group
PI(16:0/18:0)
****
3e+07
2e+07
1e+07
0e+00
Expression
D
CK
Group

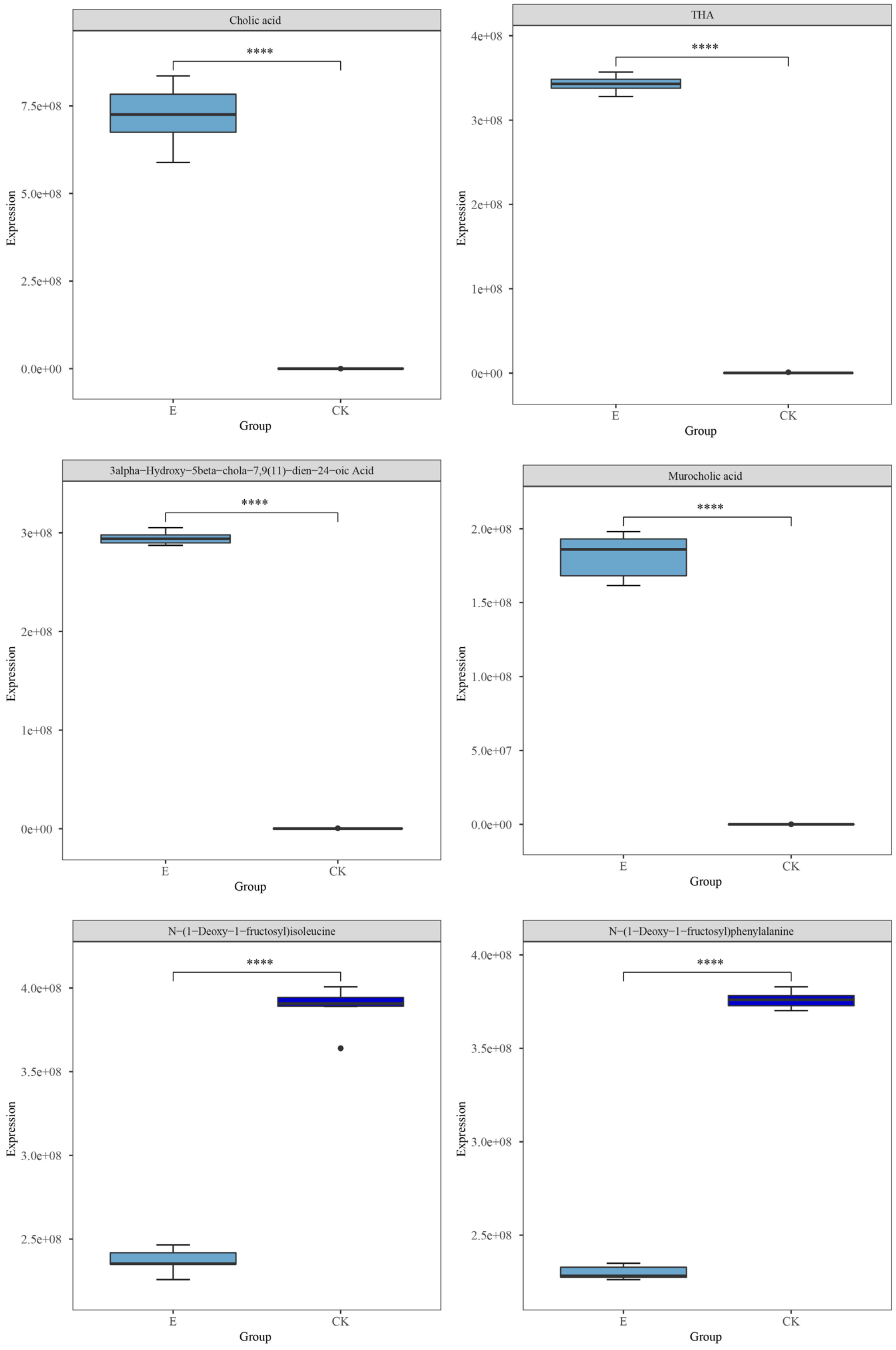
Cholic acid
****
7.5e+08
5.0e+08
2.5e+08
0.0e+00
Expression
E
CK
Group
THA
****
4e+08
3e+08
2e+08
1e+08
0e+00
Expression
E
CK
Group
3alpha−Hydroxy−5beta−chola−7,9(11)−dien−24−oic Acid
****
3e+08
2e+08
1e+08
0e+00
Expression
E
CK
Group
Murocholic acid
****
2.0e+08
1.5e+08
1.0e+08
5.0e+07
0.0e+00
Expression
E
CK
Group
N−(1−Deoxy−1−fructosyl)isoleucine
****
4.0e+08
3.5e+08
3.0e+08
2.5e+08
Expression
E
CK
Group
N−(1−Deoxy−1−fructosyl)phenylalanine
****
4.0e+08
3.5e+08
3.0e+08
2.5e+08
Expression
E
CK
Group

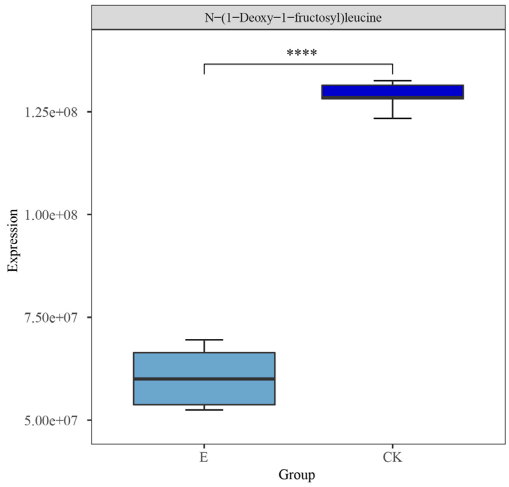
N−(1−Deoxy−1−fructosyl)leucine
****
1.25e+08
1.00e+08
7.50e+07
5.00e+07
Expression
E
CK
Group

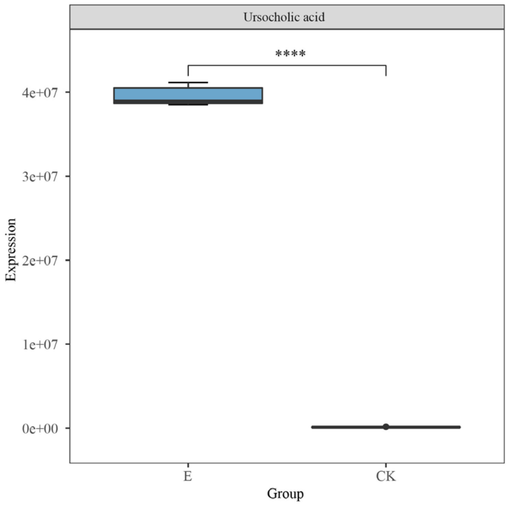
Ursocholic acid
****
4e+07
3e+07
2e+07
1e+07
0e+00
Expression
E
CK
Group

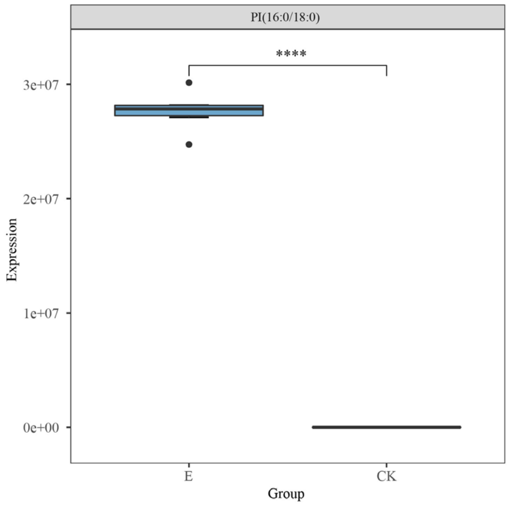
PI(16:0/18:0)
****
3e+07
2e+07
1e+07
0e+00
Expression
E
CK
Group

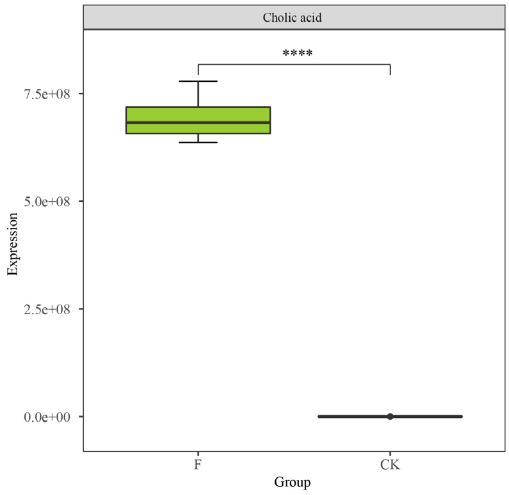
Cholic acid
****
7.5e+08
5.0e+08
2.5e+08
0.0e+00
Expression
F
CK
Group

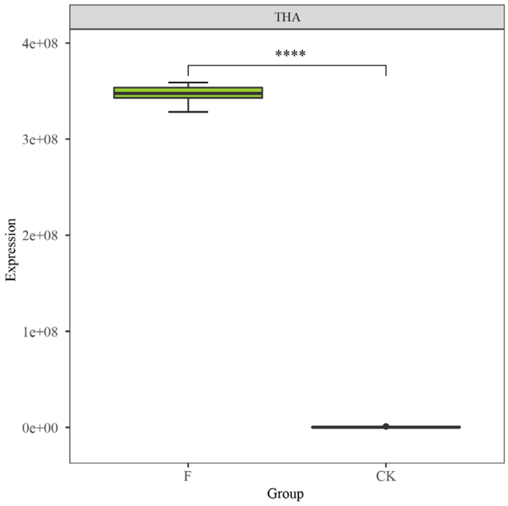
THA
****
4e+08
3e+08
2e+08
1e+08
0e+00
Expression
F
CK
Group

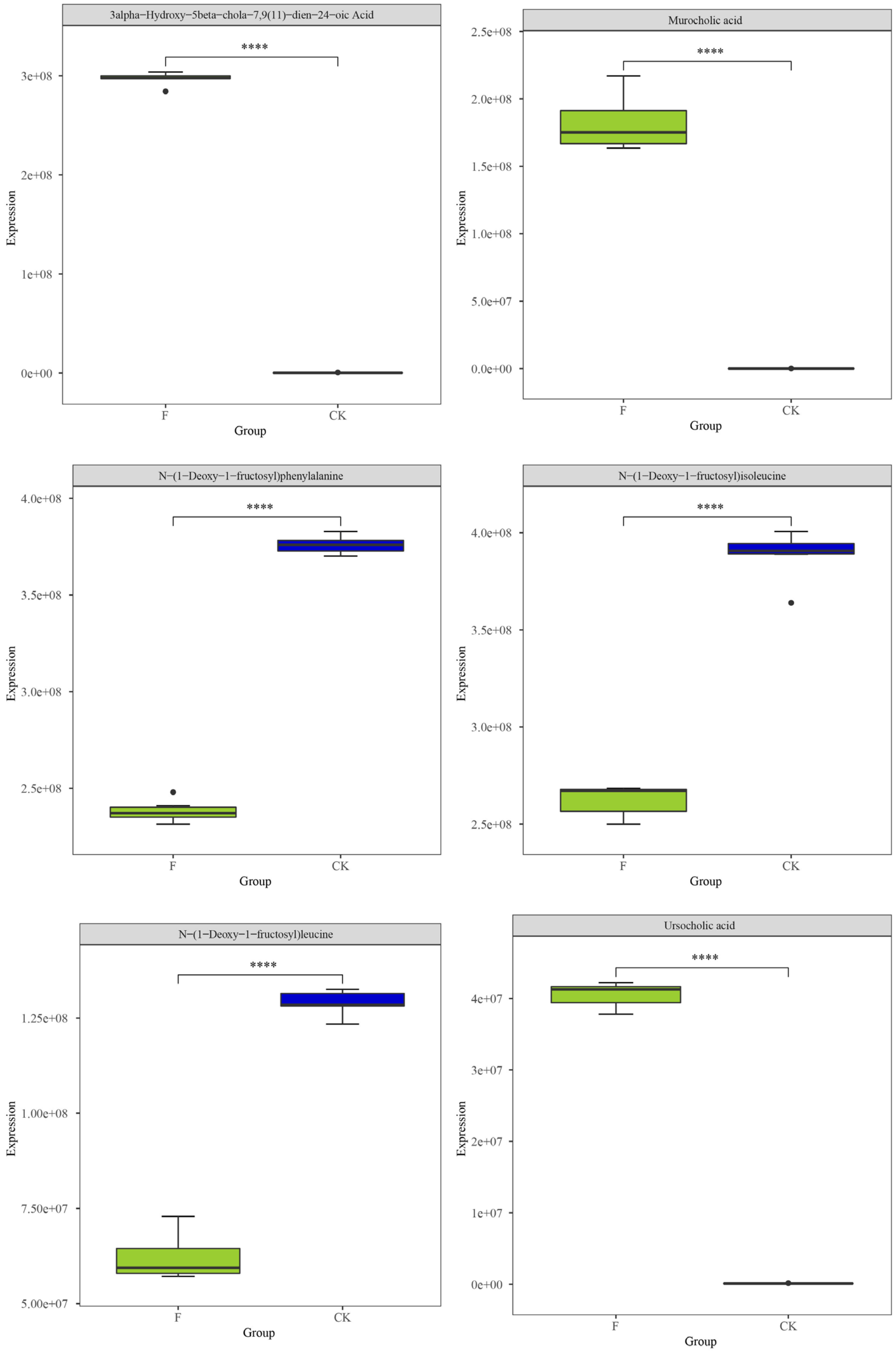
3alpha−Hydroxy−5beta−chola−7,9(11)−dien−24−oic Acid
Murocholic acid
N−(1−Deoxy−1−fructosyl)phenylalanine
N−(1−Deoxy−1−fructosyl)isoleucine
N−(1−Deoxy−1−fructosyl)leucine
Ursocholic acid
****
Expression
Group
F
CK
3e+08
2e+08
1e+08
0e+00
2.5e+08
2.0e+08
1.5e+08
1.0e+08
5.0e+07
0.0e+00
4.0e+08
3.5e+08
3.0e+08
1.25e+08
1.00e+08
7.50e+07
5.00e+07
4e+07
3e+07
2e+07
1e+07

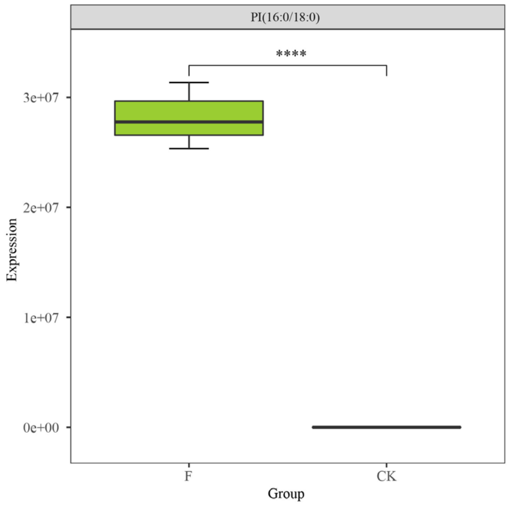
PI(16:0/18:0)
****
Expression
3e+07
2e+07
1e+07
0e+00
F
CK
Group

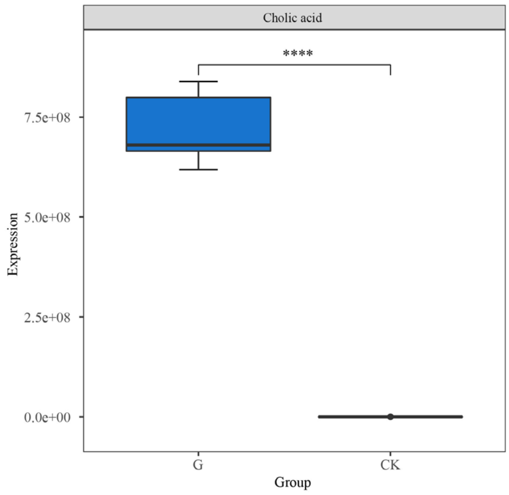
Cholic acid
****
Expression
7.5e+08
5.0e+08
2.5e+08
0.0e+00
G
CK
Group

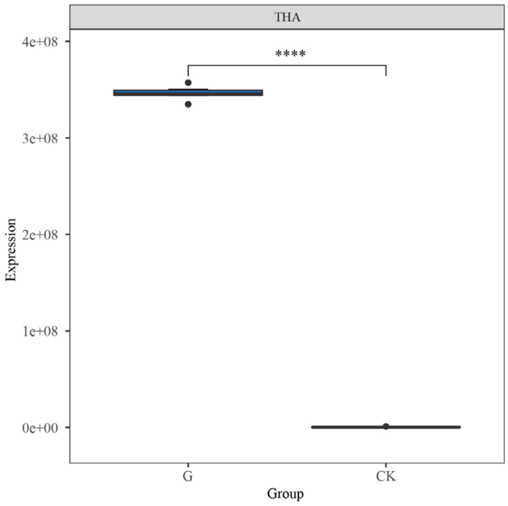
THA
****
Expression
4e+08
3e+08
2e+08
1e+08
0e+00
G
CK
Group

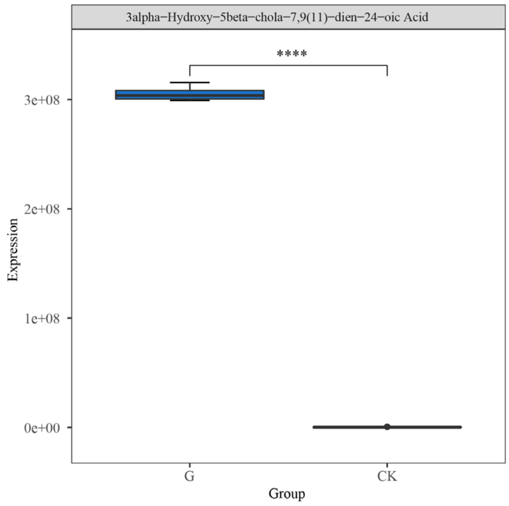
3alpha−Hydroxy−5beta−chola−7,9(11)−dien−24−oic Acid
****
Expression
3e+08
2e+08
1e+08
0e+00
G
CK
Group

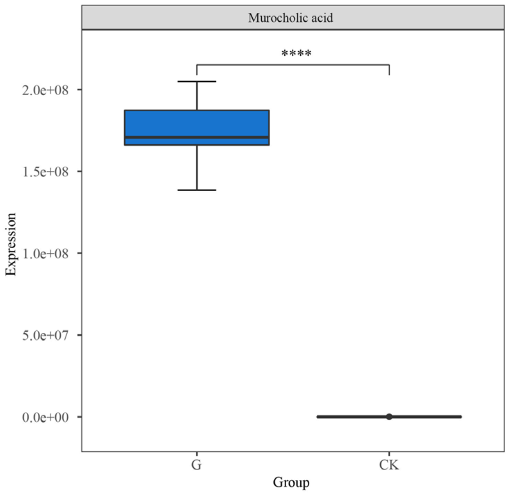
Murocholic acid
****
Expression
2.0e+08
1.5e+08
1.0e+08
5.0e+07
0.0e+00
G
CK
Group

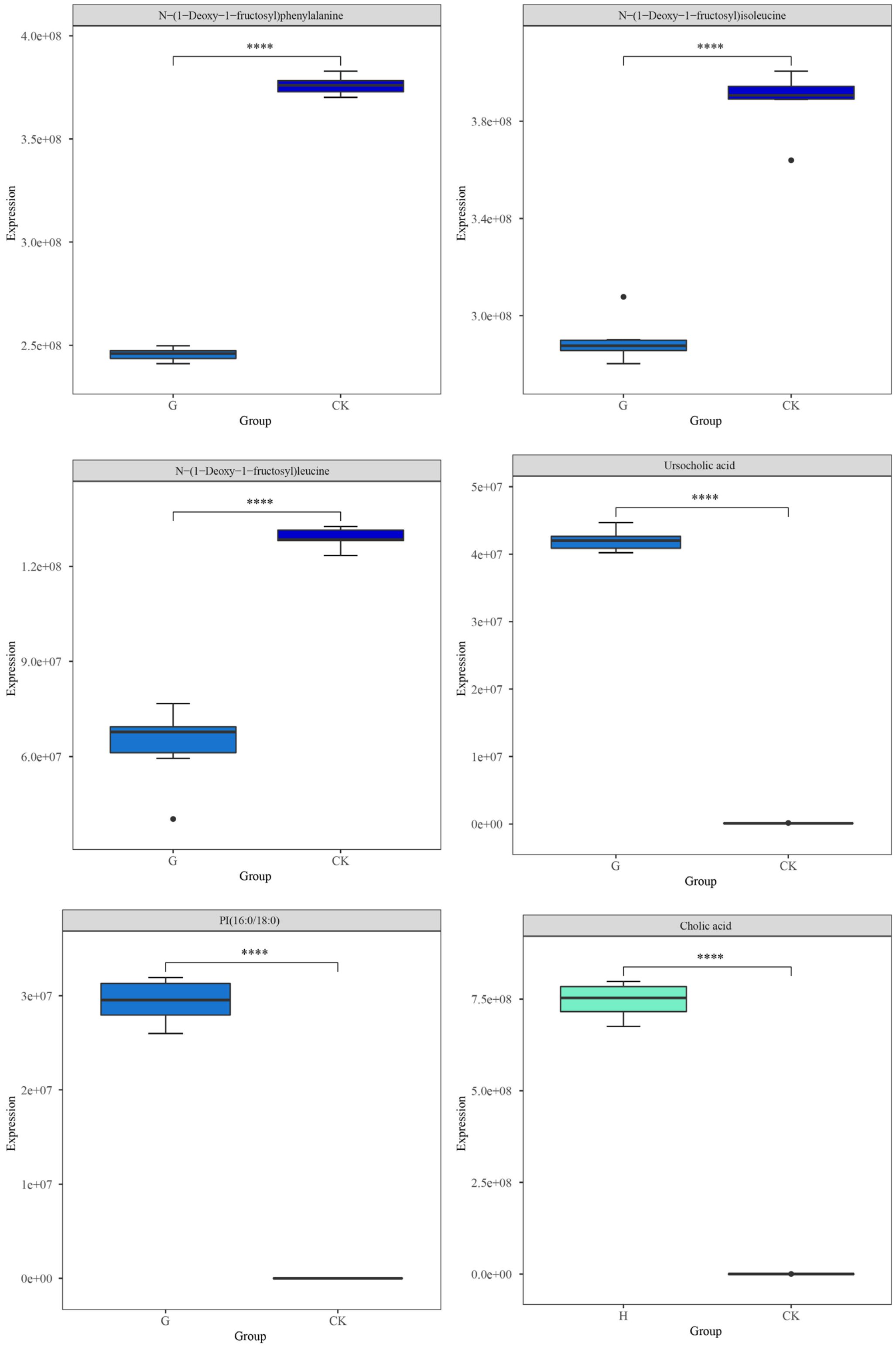
N−(1−Deoxy−1−fructosyl)phenylalanine
4.0e+08
3.5e+08
3.0e+08
2.5e+08
Expression
****
G
CK
Group
N−(1−Deoxy−1−fructosyl)isoleucine
3.8e+08
3.4e+08
3.0e+08
Expression
****
G
CK
Group
N−(1−Deoxy−1−fructosyl)leucine
1.2e+08
9.0e+07
6.0e+07
Expression
****
G
CK
Group
Ursocholic acid
5e+07
4e+07
3e+07
2e+07
1e+07
0e+00
Expression
****
G
CK
Group
PI(16:0/18:0)
3e+07
2e+07
1e+07
0e+00
Expression
****
G
CK
Group
Cholic acid
7.5e+08
5.0e+08
2.5e+08
0.0e+00
Expression
****
H
CK
Group

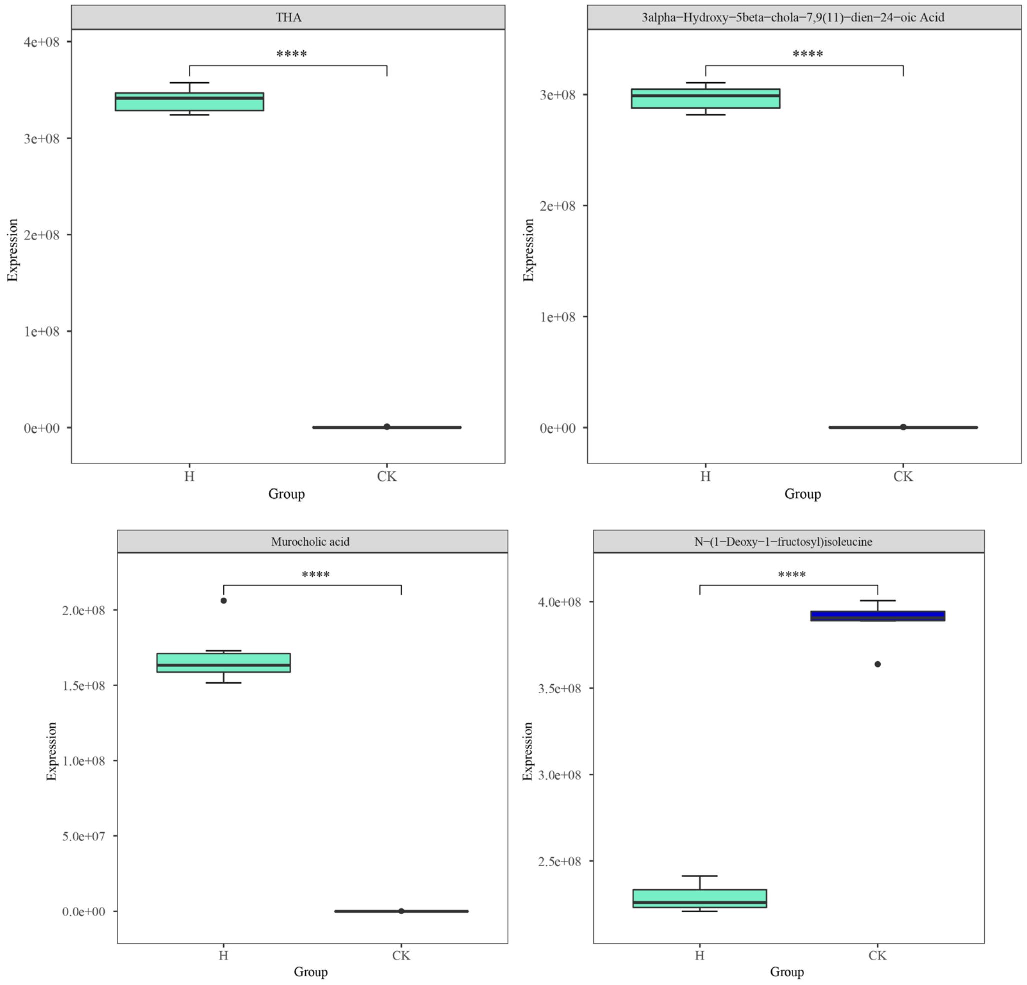
THA
****
4e+08
3e+08
2e+08
1e+08
0e+00
Expression
H
CK
Group
3alpha−Hydroxy−5beta−chola−7,9(11)−dien−24−oic Acid
****
3e+08
2e+08
1e+08
0e+00
Expression
H
CK
Group
Murocholic acid
****
2.0e+08
1.5e+08
1.0e+08
5.0e+07
0.0e+00
Expression
H
CK
Group
N−(1−Deoxy−1−fructosyl)isoleucine
****
4.0e+08
3.5e+08
3.0e+08
2.5e+08
Expression
H
CK
Group

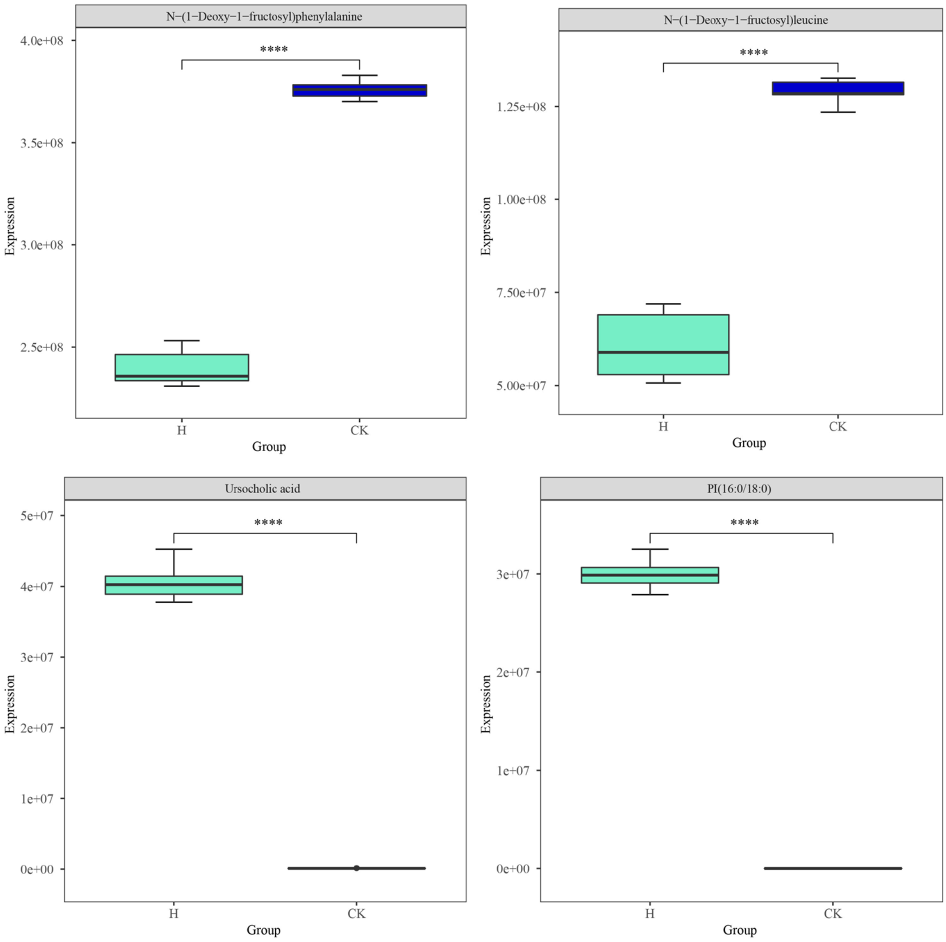

Figure S6. Distribution of crucial DEMs between Groups vs. CK sample comparison group

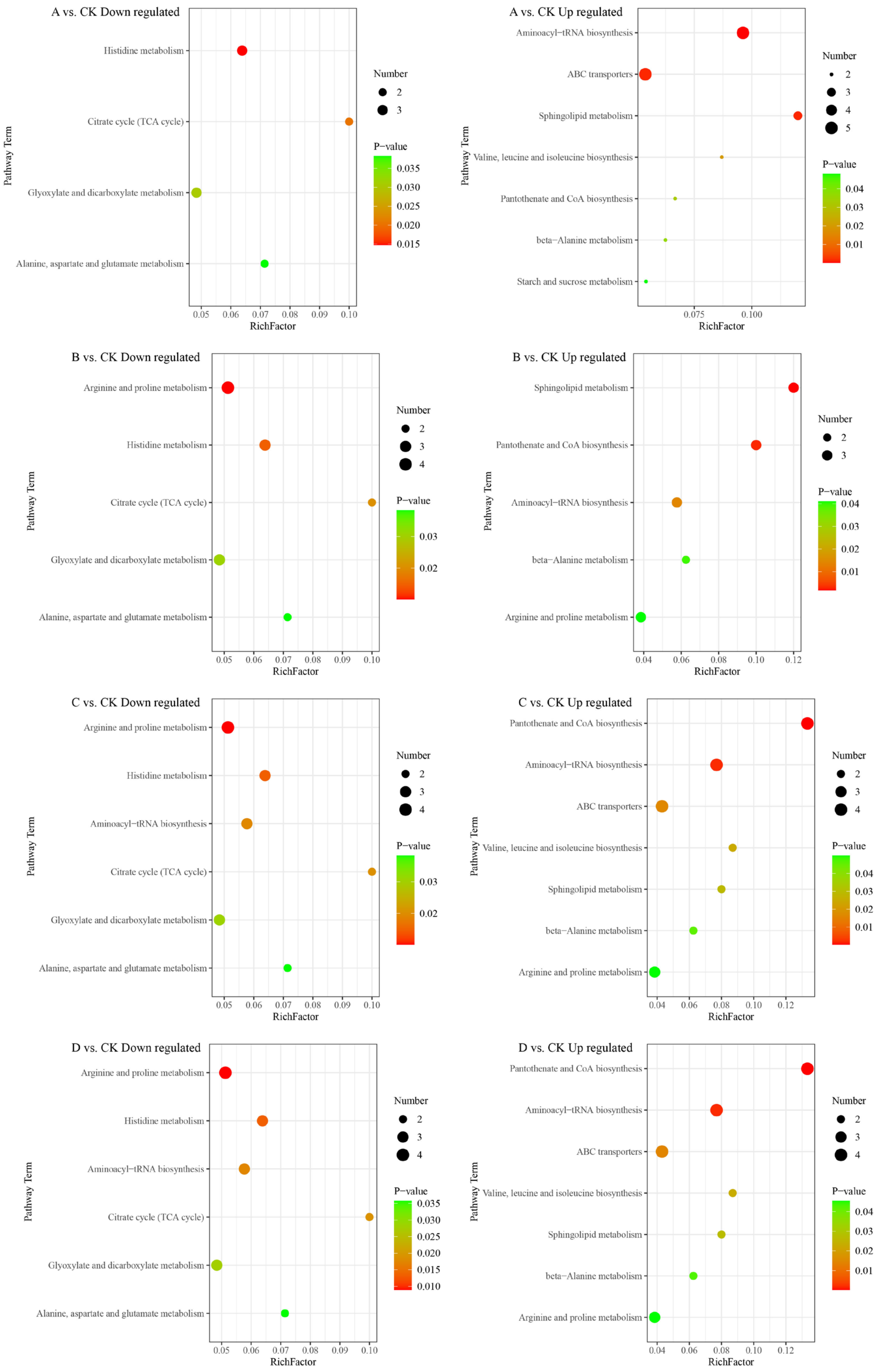
A vs. CK Down regulated
Histidine metabolism
Citrate cycle (TCA cycle)
Glyoxylate and dicarboxylate metabolism
Alanine, aspartate and glutamate metabolism
Pathway Term
0.05 0.06 0.07 0.08 0.09 0.10
RichFactor
Number
2
3
P−value
0.035
0.030
0.025
0.020
0.015
A vs. CK Up regulated
Aminoacyl−tRNA biosynthesis
ABC transporters
Sphingolipid metabolism
Valine, leucine and isoleucine biosynthesis
Pantothenate and CoA biosynthesis
beta−Alanine metabolism
Starch and sucrose metabolism
Pathway Term
0.075 0.100
RichFactor
Number
2
3
4
5
P−value
0.04
0.03
0.02
0.01
B vs. CK Down regulated
Arginine and proline metabolism
Histidine metabolism
Citrate cycle (TCA cycle)
Glyoxylate and dicarboxylate metabolism
Alanine, aspartate and glutamate metabolism
Pathway Term
0.05 0.06 0.07 0.08 0.09 0.10
RichFactor
Number
2
3
4
P−value
0.03
0.02
B vs. CK Up regulated
Sphingolipid metabolism
Pantothenate and CoA biosynthesis
Aminoacyl−tRNA biosynthesis
beta−Alanine metabolism
Arginine and proline metabolism
Pathway Term
0.04 0.06 0.08 0.10 0.12
RichFactor
Number
2
3
P−value
0.04
0.03
0.02
0.01
C vs. CK Down regulated
Arginine and proline metabolism
Histidine metabolism
Aminoacyl−tRNA biosynthesis
Citrate cycle (TCA cycle)
Glyoxylate and dicarboxylate metabolism
Alanine, aspartate and glutamate metabolism
Pathway Term
0.05 0.06 0.07 0.08 0.09 0.10
RichFactor
Number
2
3
4
P−value
0.03
0.02
C vs. CK Up regulated
Pantothenate and CoA biosynthesis
Aminoacyl−tRNA biosynthesis
ABC transporters
Valine, leucine and isoleucine biosynthesis
Sphingolipid metabolism
beta−Alanine metabolism
Arginine and proline metabolism
Pathway Term
0.04 0.06 0.08 0.10 0.12
RichFactor
Number
2
3
4
P−value
0.04
0.03
0.02
0.01
D vs. CK Down regulated
Arginine and proline metabolism
Histidine metabolism
Aminoacyl−tRNA biosynthesis
Citrate cycle (TCA cycle)
Glyoxylate and dicarboxylate metabolism
Alanine, aspartate and glutamate metabolism
Pathway Term
0.05 0.06 0.07 0.08 0.09 0.10
RichFactor
Number
2
3
4
P−value
0.035
0.030
0.025
0.020
0.015
0.010
D vs. CK Up regulated
Pantothenate and CoA biosynthesis
Aminoacyl−tRNA biosynthesis
ABC transporters
Valine, leucine and isoleucine biosynthesis
Sphingolipid metabolism
beta−Alanine metabolism
Arginine and proline metabolism
Pathway Term
0.04 0.06 0.08 0.10 0.12
RichFactor
Number
2
3
4
P−value
0.04
0.03
0.02
0.01

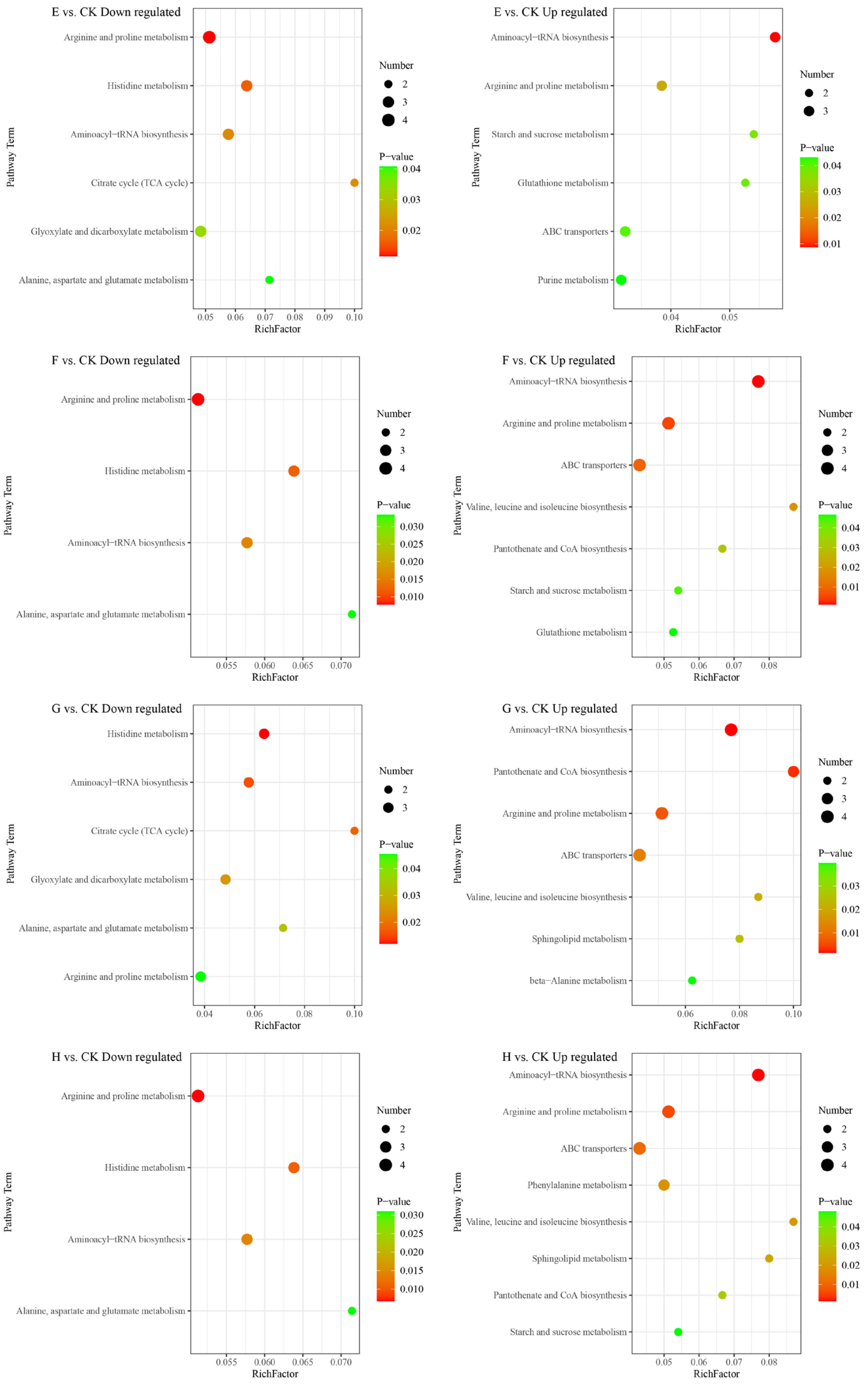

Figure S7. DEM enrichment in KEGG pathways

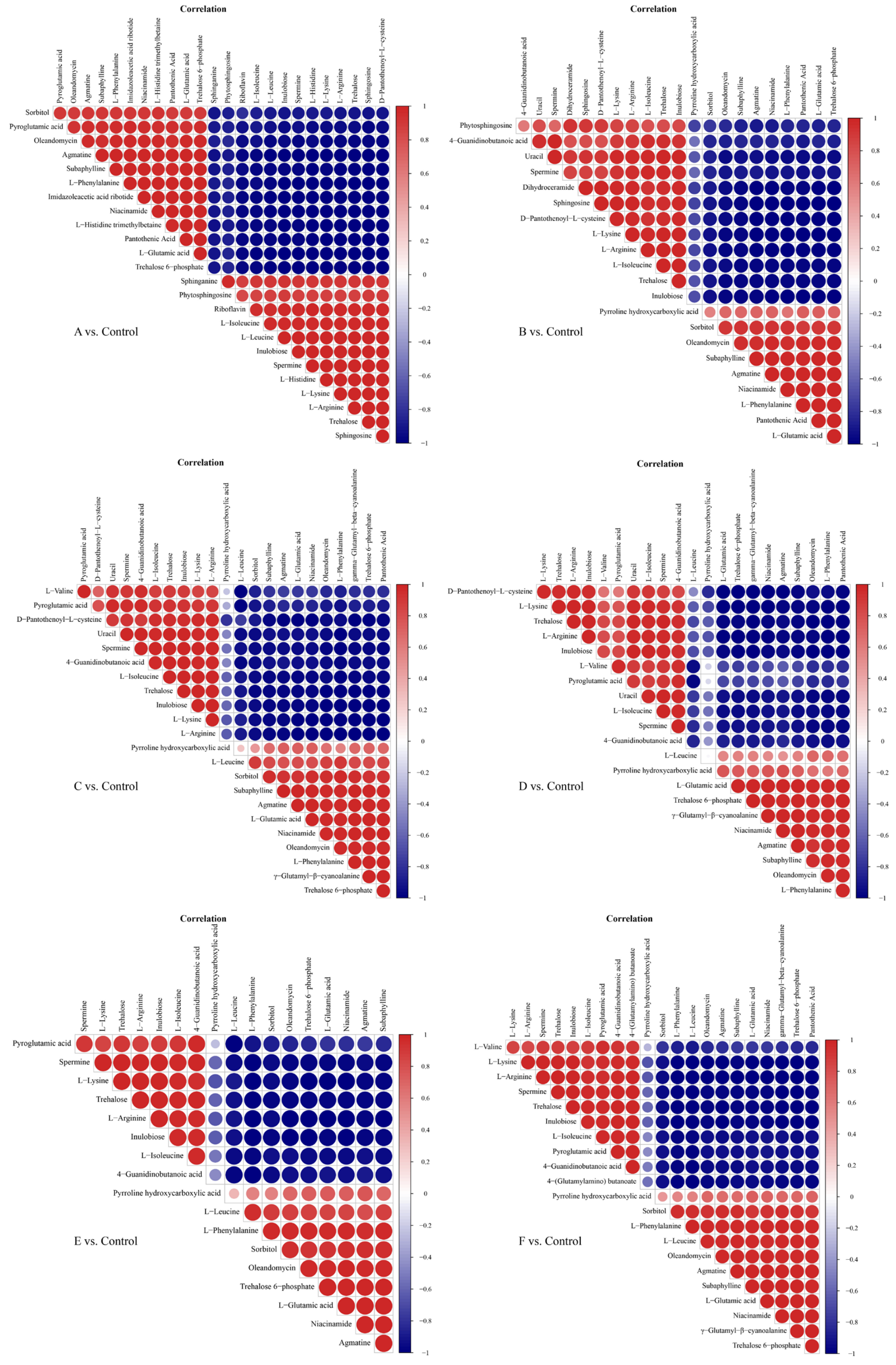
Correlation
A vs. Control
Correlation
B vs. Control
Correlation
C vs. Control
Correlation
D vs. Control
Correlation
E vs. Control
Correlation
F vs. Control

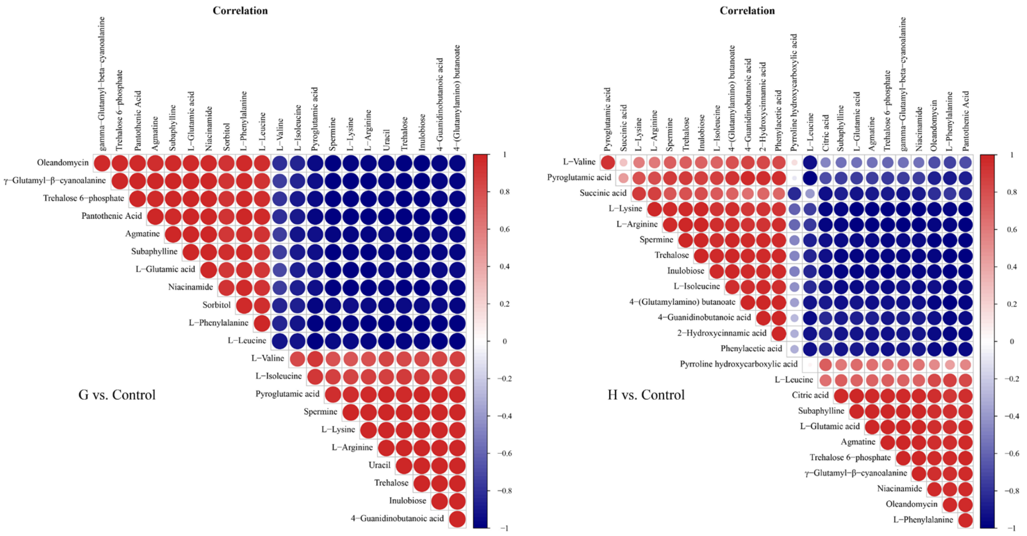

Figure S8. DEM correlation plots
